# Supplementary material for: RNA m6A methylation regulates sorafenib resistance in liver cancer through FOXO3‐mediated autophagy
Source: EMBO J. 2020 May 5;39(12):e103181. doi: 10.15252/embj.2019103181 (PMC7298296; doi:10.15252/embj.2019103181)

APPENDIX

Table of Contents

Appendix Figure S1: **Hypoxia condition frequently occurred in HCC.**

Appendix Figure S2: **Knockdown of METTL3 enhanced chemo-resistance in HCC.**

Appendix Figure S3: **Regulation of autophagy by METTL3.**

Appendix Figure S4: **Regulation of autophagy by METTL3.**

Appendix Figure S5: **FOXO3 mediated autophagy in HCC.**

APPENDIX FIGURE LEGENDS

Appendix Figure S1. Hypoxia condition frequently occurred in HCC.

A The Volcano plot of gene expression changes in the transcriptome profile of sorafenib-sensitive liver tumors and sorafenib-resistant liver tumors.

B METTL3 down-regulation was significantly associated with shorter overall survival in HCC in TCGA (setting gate 33%).

C The mRNA expression level of HIF-1α is significantly up-regulated in liver tumors (n=225) compared to normal liver tissues (n=220) in GSE14520-GPL3921.

D The mRNA expression level of HIF-1α is significantly up-regulated in liver tumors (n=21) compared to normal liver tissues (n=22) (GSE14520-GPL57).

E The protein level of HIF-1α in SMMC-7721 cells derived xenografts in the subcutaneous implantation mice model.

F Representative images of HIF1α by IHC staining in SMMC-7721 cells derived xenografts in the subcutaneous implantation mice model. Scale bar, 200μm. Quantification of HIF1α expression in immunohistochemical images by Image Pro Plus (IPP) analysis in F-1.

G The HE staining and the protein level of HIF-1α by IHC staining in non-tumor tissue and orthotopic Bel-7402 cells derived xenografts. Scale bar, 1mm. Quantification of HIF-1α expression in immunohistochemical images by Image Pro Plus (IPP) analysis in G-1.

Data information: In all relevant panels, **p<0.01; ***p<0.001; ****p<0.0001; Two-tailed t-test. Data are presented as mean ± SD.

Appendix Figure S2. Knockdown of METTL3 enhanced chemo-resistance in HCC.

A Knockdown of METTL3 in WRL68 cells by lentiviral shRNAs. The knockdown effect was verified at the protein levels by Western blot.

B Overexpression of METTL3 in WRL68 cells and verified by Western blot.

C Sensitivity of WRL68 with various levels of METTL3 to sorafenib treatment for 24 h.

D Cell proliferation in WRL68 with various levels of METTL3 by CKK8 assay.

E Cell proliferation in WRL68 with various levels of METTL3 by cell number counting.

F Knockdown of METTL3 in HCCs by lentiviral shRNAs. The knockdown effect was verified at the protein levels by Western blot.

G Knockdown of METTL3 in HCCs by lentiviral shRNAs. The knockdown effect was verified at the mRNA levels by RT-PCR.

H The global RNA m^6A level in METTL3-knockdown SMMC-7721, Bel-7402 and HepG-2 cells by dot-blotting assay.

I The IC50 of METTL3-knockdown HepG-2 cells after treated with sorafenib for 24h under hypoxia condition (1%O_2).

J-L Cell survival assay of METTL3-knockdown SMMC-7721 cells (J), Bel-7402 cells (K) and HepG-2 cells (L) after treated with sorafenib for 24h under hypoxia condition (1%O_2).

M-O The IC50 of METTL3-knockdown SMMC-7721 (M), Bel-7402 (N) and HepG-2 cells (O) after treated with sorafenib for 24h under normoxia condition (21%O_2).

P-Q Overexpression of wildtype METTL3 or catalytic mutant METTL3 in METTL3-knockdown SMMC-7721 cells (P) and Bel-7402 cells (Q) and verified by Western blot.

R-T Cell growth determined by CCK8 assay in METTL3-knockdown SMMC-7721 cells (R), Bel-7402 cells (S) and HepG-2 cells (T) with rescueing of shRNA-resistant wildtype METTL3 or catalytic mutant METTL3.

U The relative mRNA expression levels of angiogenesis genes were detected in HepG-2 cells with METTL3-knockdown by RT-PCR assay.

Data information: In all relevant panels, *p<0.05; **p<0.01; ***p<0.001; ****p<0.0001; Two-tailed t-test. Data are presented as mean ± SD and are representative of 3 independent experiments.

Appendix Figure S3. Regulation of autophagy by METTL3.

A Representative immunostaining images of LC3 in METTL3-knockdown SMMC-7721 cells, METTL3-knockdown Bel-7402 cells, naïve and sorafenib-resistant HepG-2 cells under normoxia condition (21% O_2) for 48h. Scal bar, 200μm.

B The quantification of the numbers of GFP-LC3 puncta/cell by Imaris.

C-D Overexpression of shRNA-resistant wild type METTL3 but not catalytic mutant METTL3

rescue autophagy phenotype in METTL3-knockdown SMMC-7721 cells, Bel-7402 cells (C) and sorafenib-resistant HepG-2 (D) by representative immunostaining images of LC3 under hypoxia condition for 48h. Scale bar, 200μm. The DAPI images were shown as uncombined as in Fig. 3F-H.

E Protein levels of LC3 I/II in HCCs treated with autophagy inhibitor, 3-Methyladenine (3-MA).

Data information: In all relevant panels, *$p<0.05$; **$p<0.01$; Two-tailed t-test. Data are presented as mean ± SD.

Appendix Figure S4. Regulation of autophagy by METTL3.

A Heatmap of differentially expressed genes involved in autophagy signaling pathway in METTL3-knockdown HepG-2, SMMC-7721 and Bel-7402 cells under hypoxia (1%O_2). Red color indicates up-regulated genes, while blue color indicates down-regulated genes.

B String analysis shows the connection between FOXO3 and autophagic factors.

C RNA level of FOXO3 in METTL3-knockdown HCCs under hypoxia for 48h.

D Protein level of FOXO3 in METTL3-knockdown SMMC-7721 cells with rescue of wild-type METTL3 and mutant METTL3 under hypoxia for 48h.

E Protein level of FOXO3 in METTL3-knockdown Bel-7402 cells with rescue of wild-type METTL3 and mutant METTL3 under hypoxia for 48h.

F Protein level of FOXO3 in sorafenib-resistant HepG-2 cells with rescue of wild-type METTL3 and mutant METTL3 under hypoxia for 48h.

G Polysome profiling analyzed in METTL3-knockdown Bel-7402 cells under hypoxia for 48h.

H Metagene analysis of m^6A in FOXO3 at a spring-loaded base modification (GSE65380).

I The m^6A level in FOXO3 mRNA in METTL3-knockdown Bel-7402 cells with rescue of wild-type METTL3 and mutant METTL3 by the YTHDF1-RIP analysis.

Data information: In all relevant panels, ****$p<0.0001$; Two-tailed t-test. Data are presented as mean ± SD and are representative of 3 independent experiments.

Appendix Figure S5. FOXO3 mediated autophagy in HCC.

A Inducible overexpression of FOXO3 in MCF-7 cells regulate biomarker genes related to autophagy (GSE113479).

B Inducible overexpression of FOXO3 in SH-SY5Y cells regulate biomarker genes related to

autophagy (GSE42762).

C Protein levels of LC3 I/II in FOXO3-knockdown Bel-7402 cells by Western blotting.

D Protein levels of LC3 I/II in FOXO3-knockdown HepG-2 cells by Western blotting.

E Validation of FOXO3-overexpression in SMMC-7721 cells by RT-PCR.

F Overexpression of FOXO3 in SMMC-7721 cells by Western blot.

G Stable knockout of METTL3 in Hepa1-6 cells by lentiviral METTL3 sgRNAs. The knockout effect was verified at the protein level by Western blot.

H Validation of FOXO3 overexpression in METTL3-knockdown Hepa1-6 cells by Western blot.

Data information: In all relevant panels, ****$p<0.0001$; Two-tailed t-test. Data are presented as mean ± SD and are representative of 3 independent experiments.

Appendix Figure S1

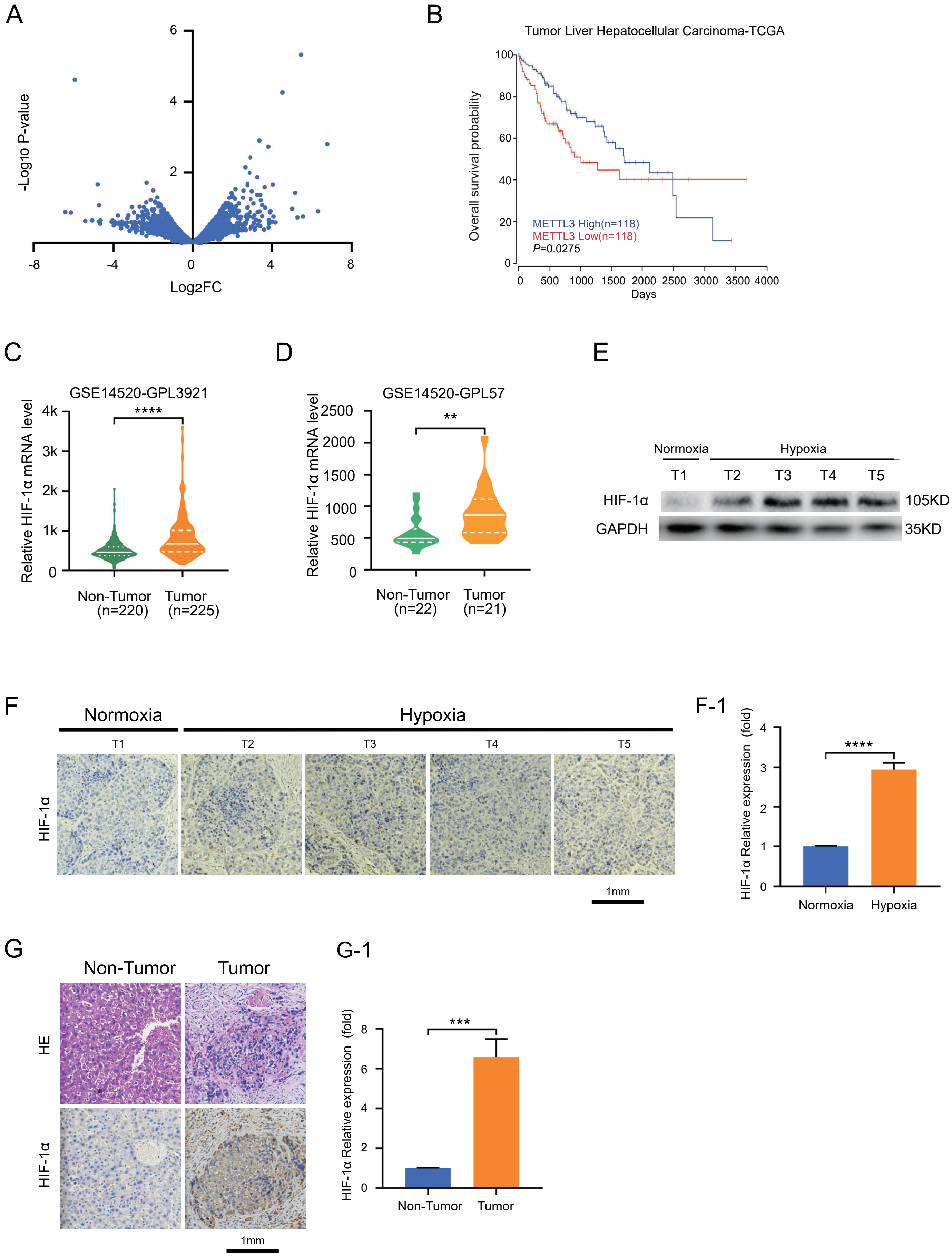

Appendix Figure S2

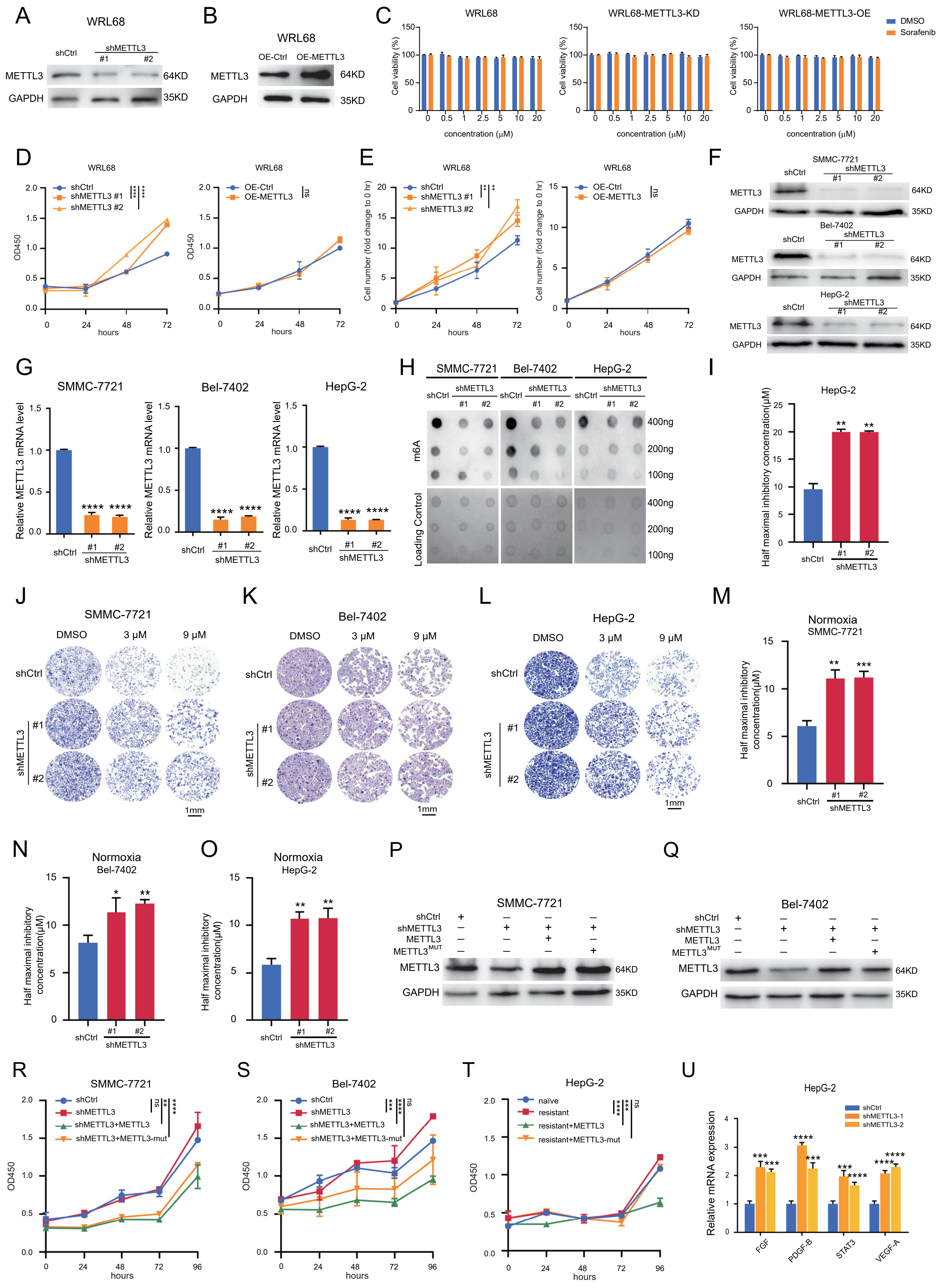

Appendix Figure S3

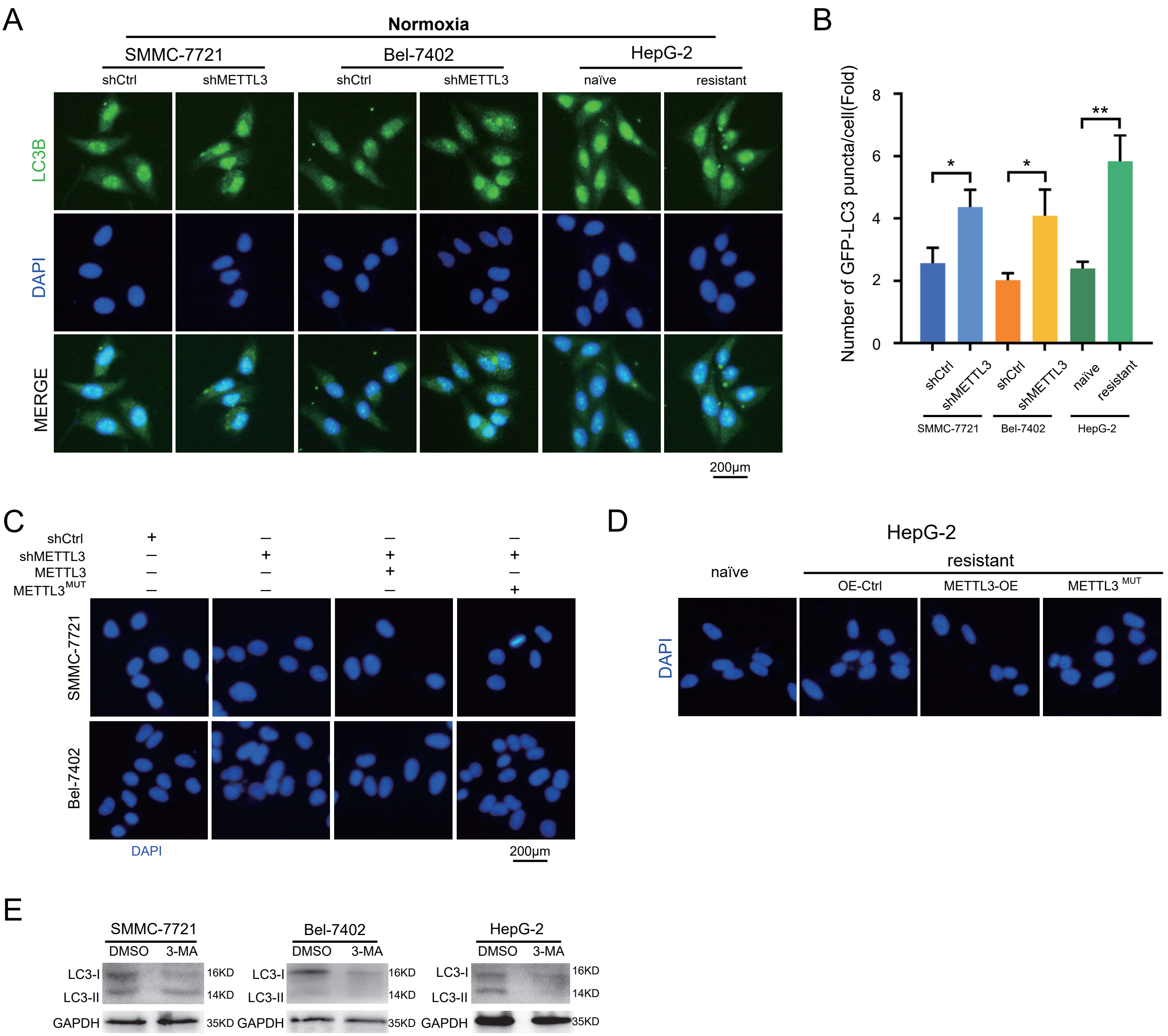

Appendix Figure S4

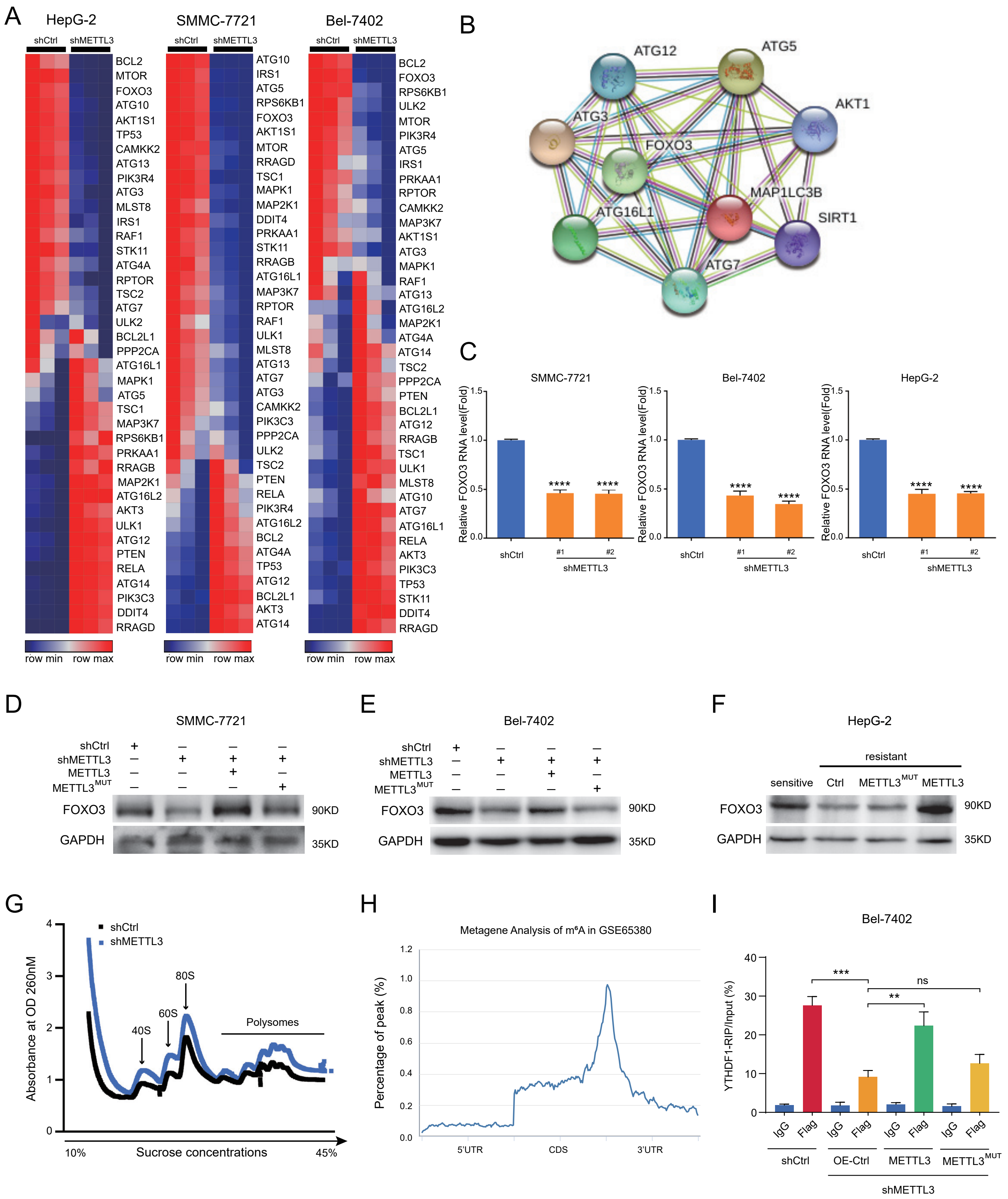

Appendix Figure S5

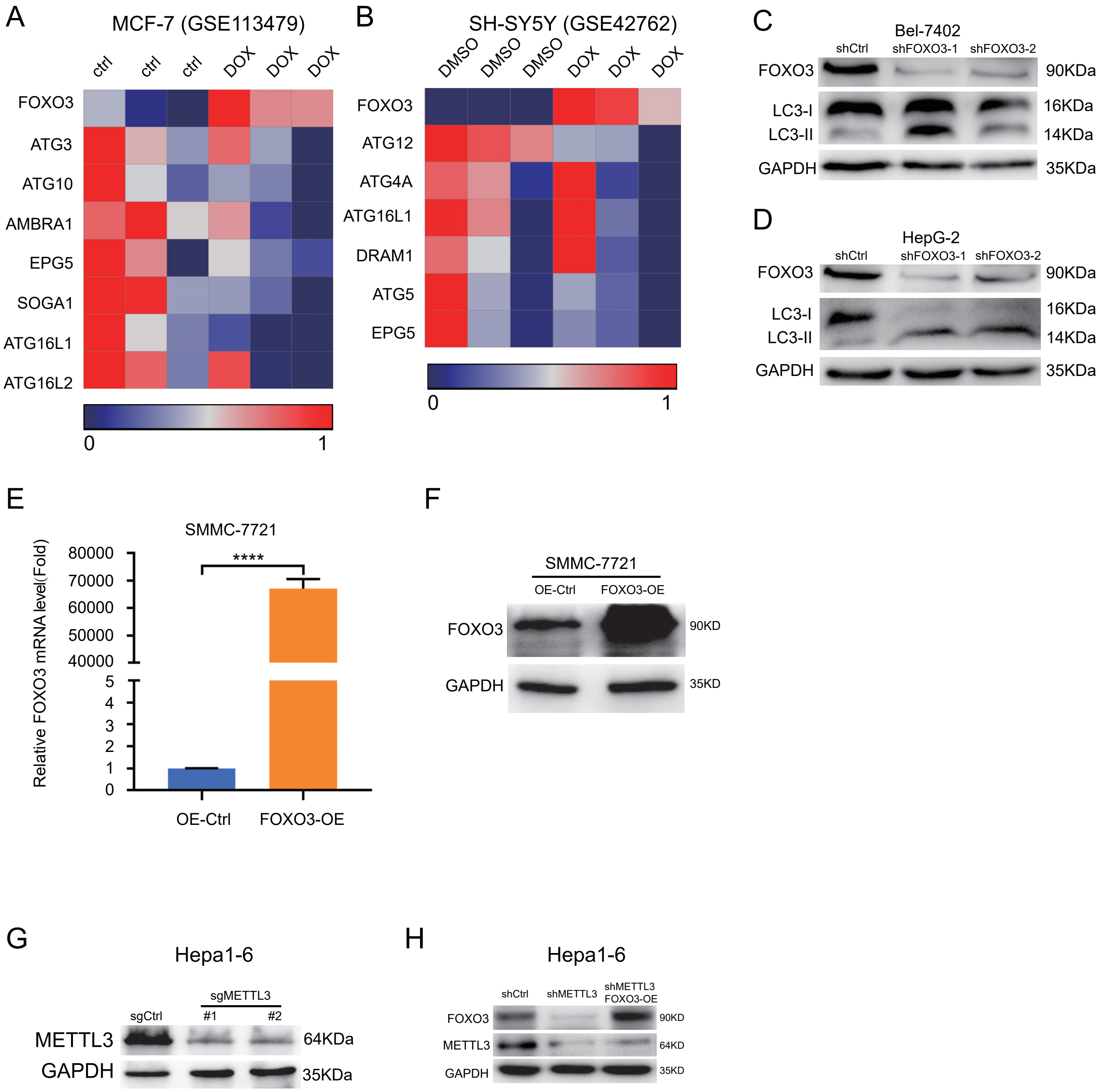

Supplement: Supplementary file 1 — Appendix [file EMBJ-39-e103181-s001.pdf]
