# Supplementary material for: RNA m6A methylation regulates sorafenib resistance in liver cancer through FOXO3‐mediated autophagy
Source: EMBO J. 2020 May 5;39(12):e103181. doi: 10.15252/embj.2019103181 (PMC7298296; doi:10.15252/embj.2019103181)

Appendix Figure S5C

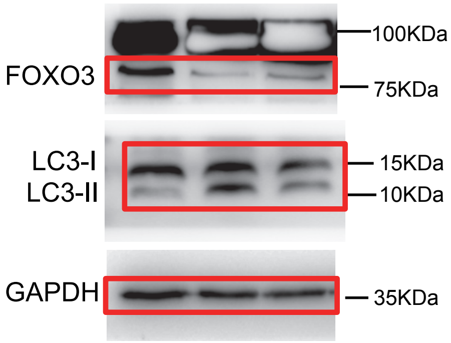

Appendix Figure S5D

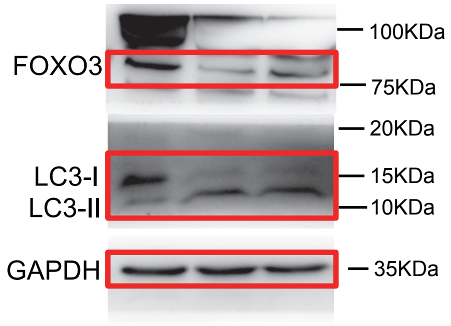

Appendix Figure S5F

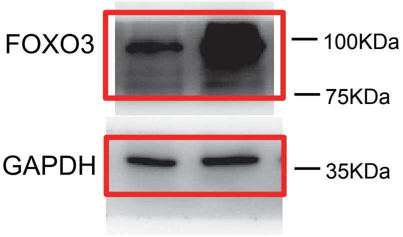

Appendix Figure S5G

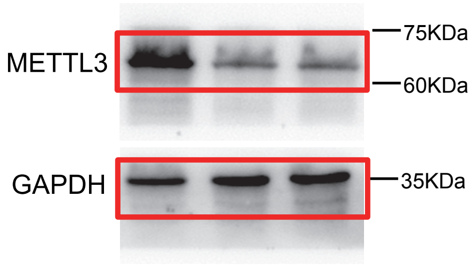

Appendix Figure S5H

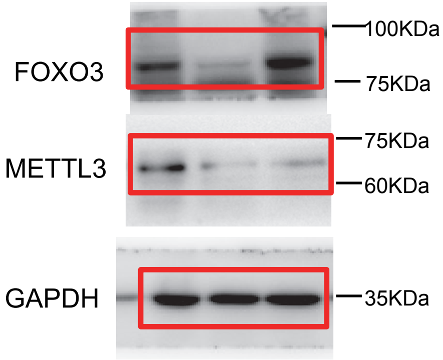

Supplement: Supplementary file 5 — Source Data for Appendix [file EMBJ-39-e103181-s010.zip › Appendix_Figure_S5.pdf]
