# Supplementary figures and images for: RNA m6A methylation regulates sorafenib resistance in liver cancer through FOXO3‐mediated autophagy
Source: EMBO J. 2020 May 5;39(12):e103181. doi: 10.15252/embj.2019103181 (PMC7298296; doi:10.15252/embj.2019103181)

Appendix Figure S4D

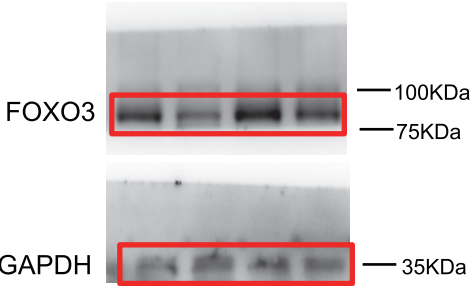

Appendix Figure S4E

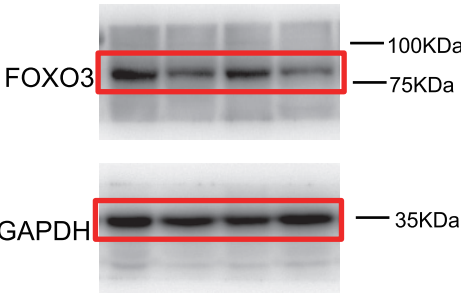

Appendix Figure S4F

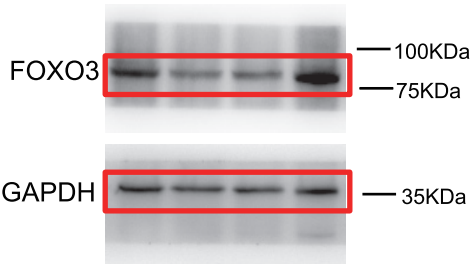

Supplement: Supplementary file 5 — Source Data for Appendix [file EMBJ-39-e103181-s010.zip › Appendix_Figure_S4.pdf]

Appendix Figure S3E

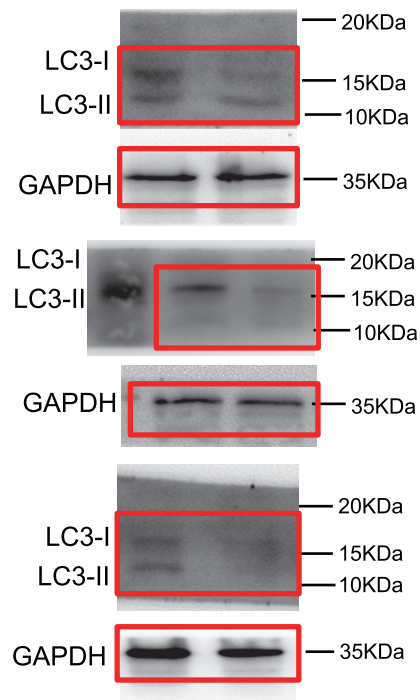

Supplement: Supplementary file 5 — Source Data for Appendix [file EMBJ-39-e103181-s010.zip › Appendix_Figure_S3.pdf]

## Appendix Figure S1D

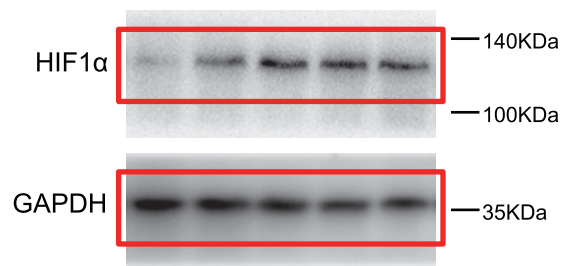

Supplement: Supplementary file 5 — Source Data for Appendix [file EMBJ-39-e103181-s010.zip › Appendix_Figure_S1.pdf]

Figure1H

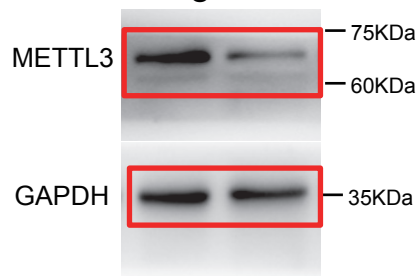

Figure1I

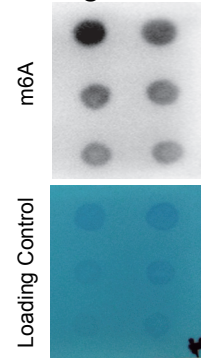

Supplement: Supplementary file 7 — Source Data for Figure 1 [file EMBJ-39-e103181-s005.pdf]

Figure2E

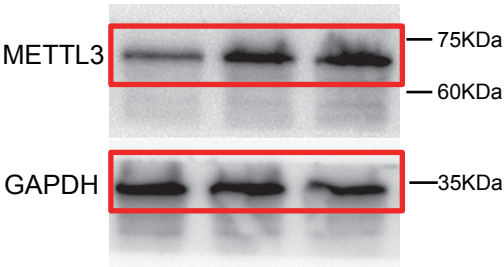

Figure2F

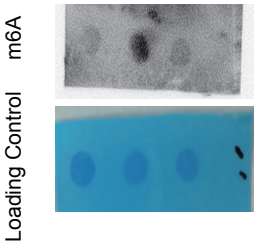

Figure2N

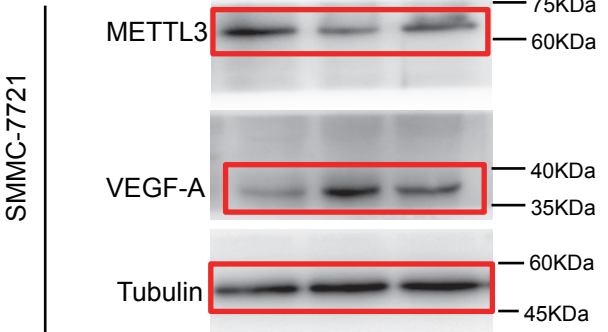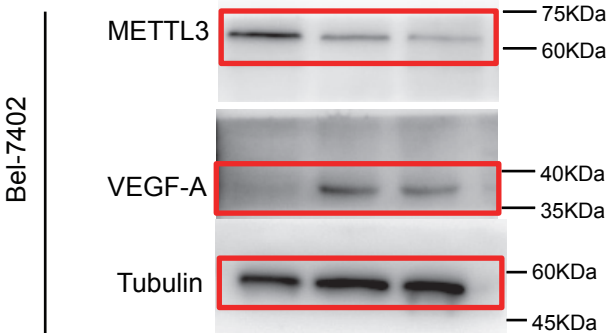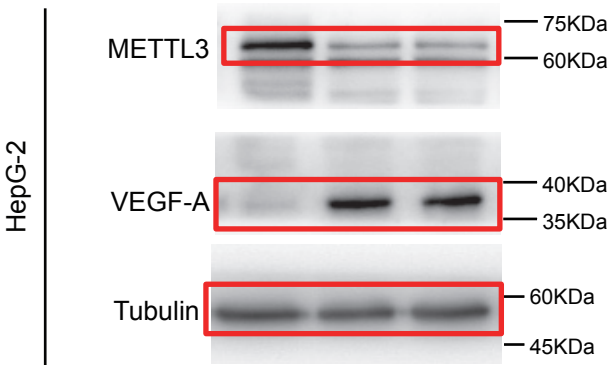

Figure2O

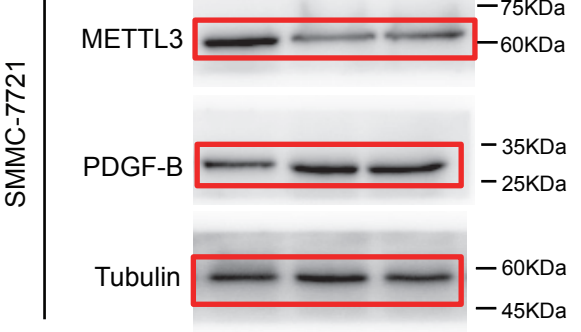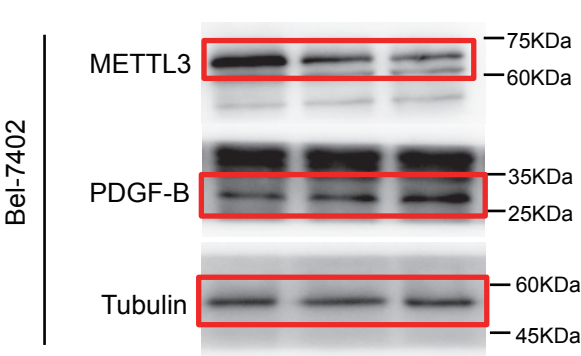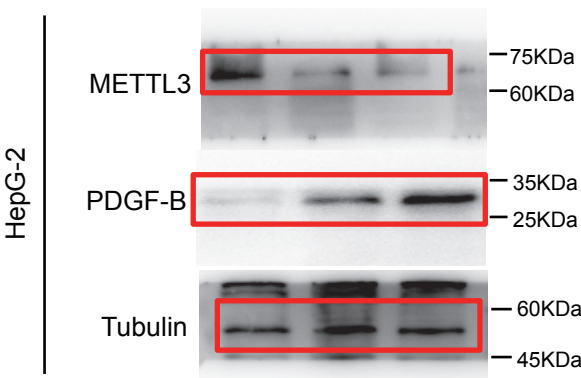

Supplement: Supplementary file 8 — Source Data for Figure 2 [file EMBJ-39-e103181-s006.pdf]

Figure3B

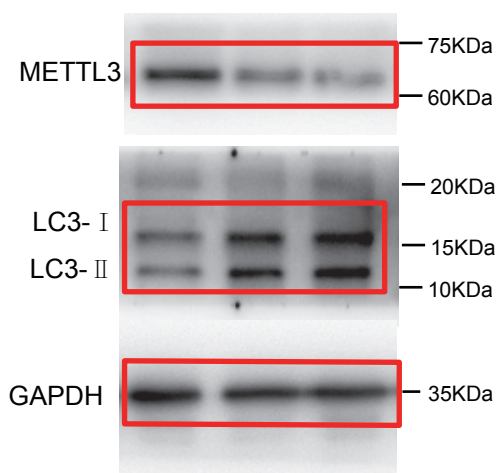

Figure3C

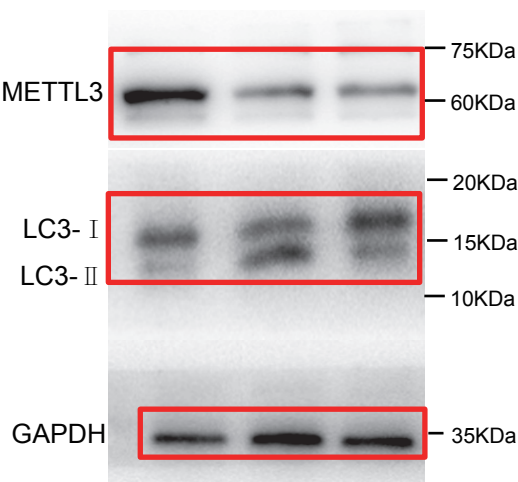

Figure3D

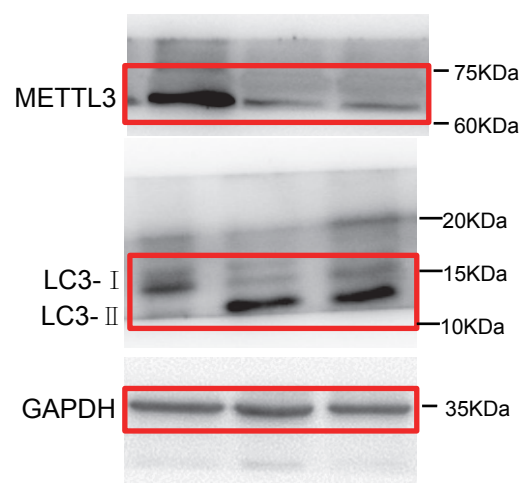

Figure3E

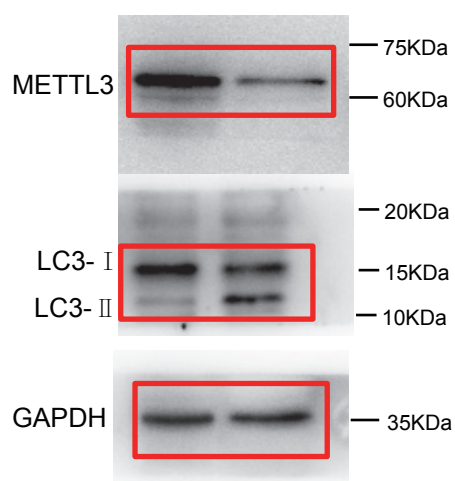

Figure3I

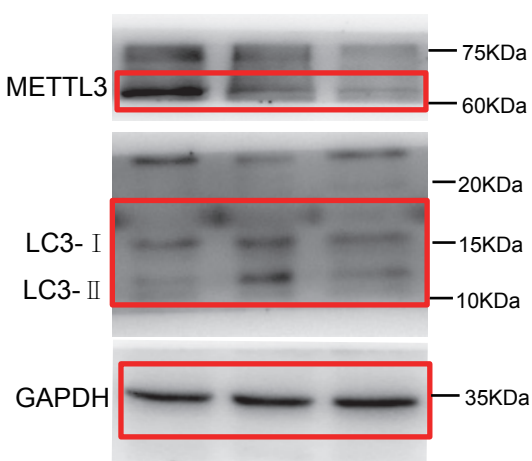

Figure3J

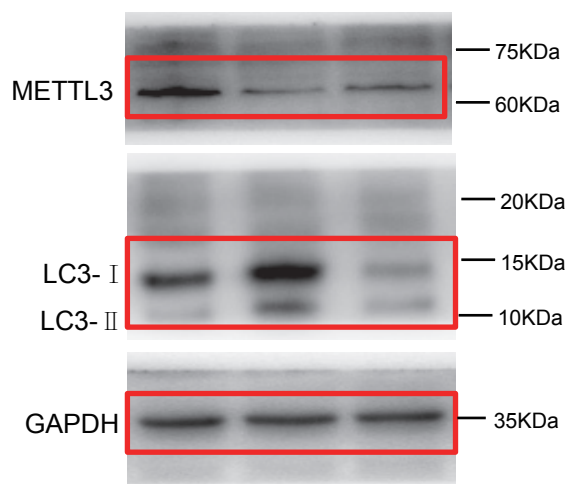

Figure3K

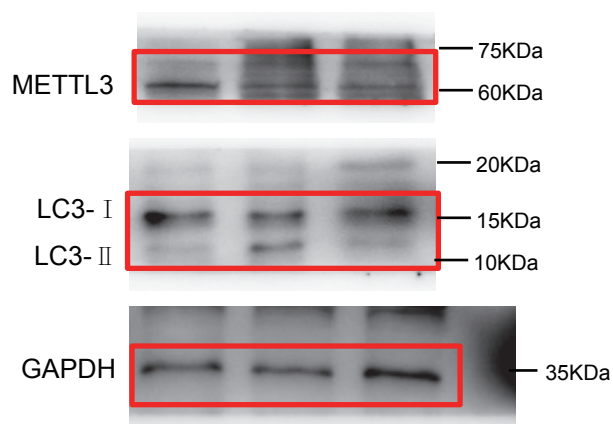

Supplement: Supplementary file 9 — Source Data for Figure 3 [file EMBJ-39-e103181-s007.pdf]

Figure4C

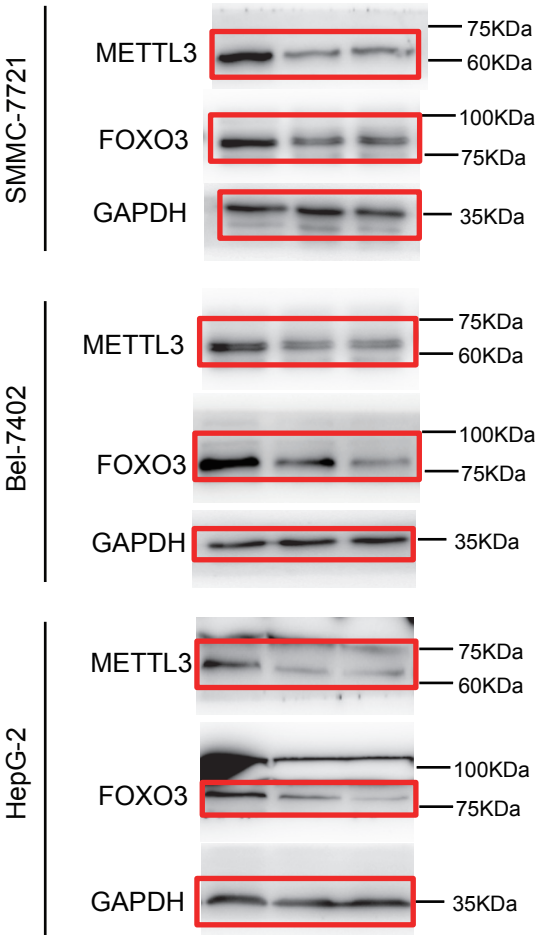

Figure4E

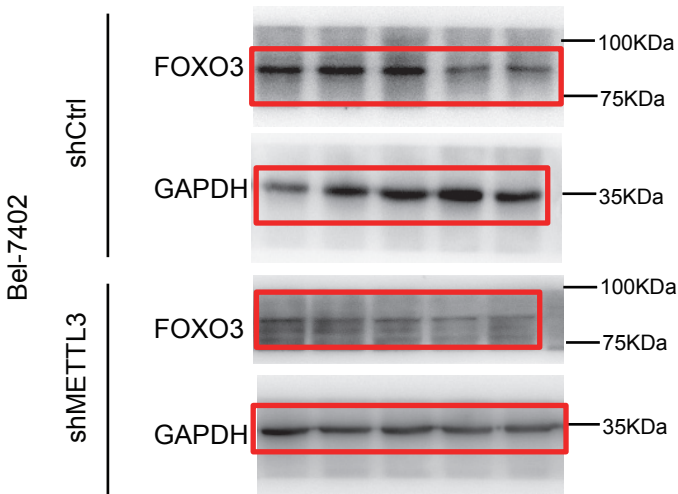

Figure4G

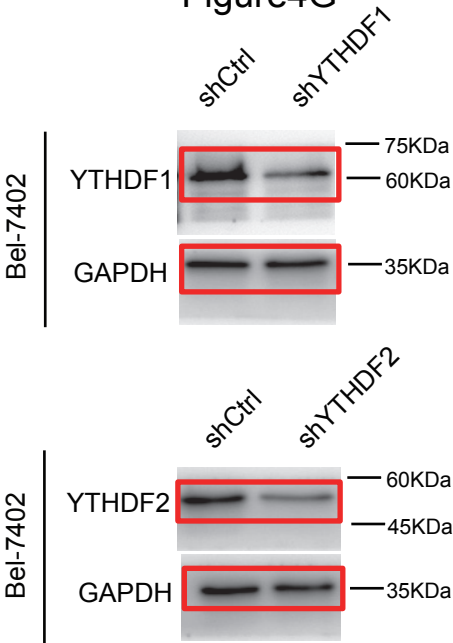

Figure4H

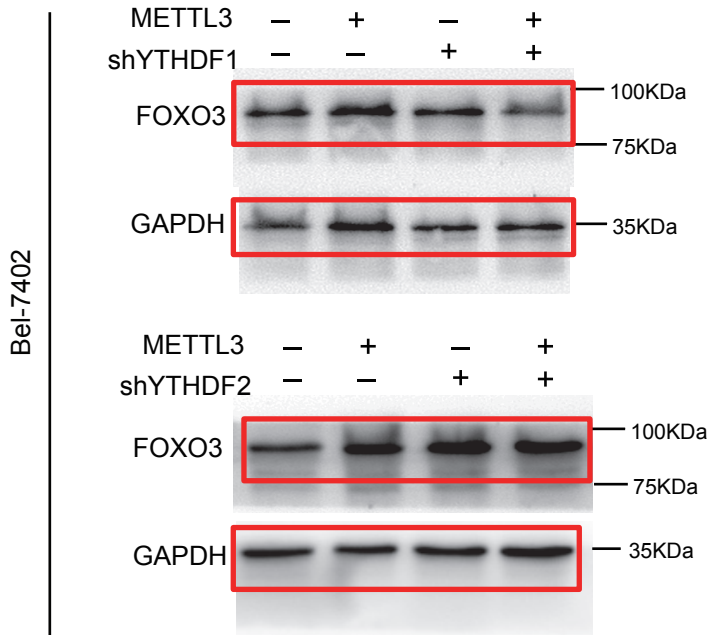

Supplement: Supplementary file 10 — Source Data for Figure 4 [file EMBJ-39-e103181-s008.pdf]

Figure5B

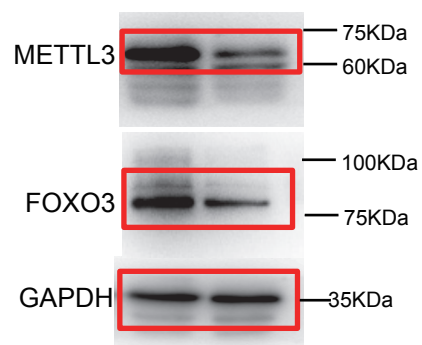

Figure5C

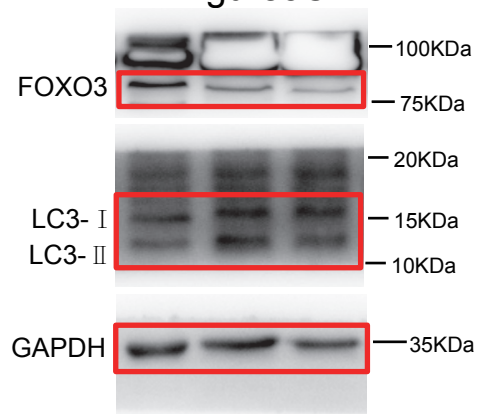

Figure5D

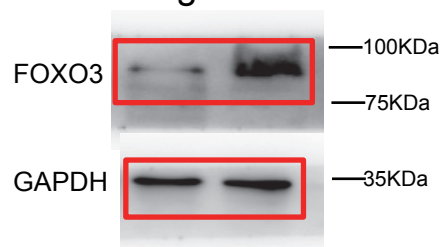

Figure5E

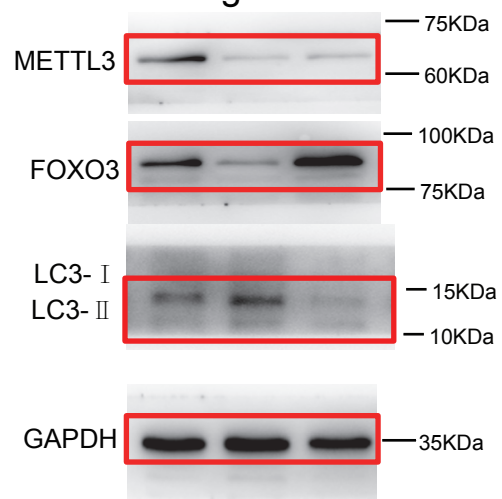

Supplement: Supplementary file 11 — Source Data for Figure 5 [file EMBJ-39-e103181-s009.pdf]
